# Generation of human-induced pluripotent stem cell-derived lung organoids for modeling respiratory syncytial virus infection and pulmonary injury

**DOI:** 10.3389/fimmu.2026.1850953

**Published:** 2026-05-28

**Authors:** Yongli Li, Yong Chen, Naiwen Cui, Xiaoli Du, Daishun Liu, Fuxun Yu

**Affiliations:** 1 Medical College, Guizhou University, Guiyang, Guizhou, China; 2 Department of Key Laboratory, Guizhou Provincial People's Hospital, Guiyang, China; 3 NHC Key Laboratory of Pulmonary Immunological Diseases, Guizhou Provincial People’s Hospital, Guiyang, China; 4 Second Clinical Medical College, Clinical Medical College, Guizhou University of Traditional Chinese Medicine, Guiyang, China; 5 Department of Respiratory and Critical Care Medicine, Guizhou Provincial People’s Hospital, Guiyang, Guizhou, China; 6 Guizhou Provincial Key Laboratory of Pathogenesis, Prevention and Treatment of Common Chronic Diseases, Guiyang, China

**Keywords:** hiPSC, lung organoids, RSV, target cells, injury

## Abstract

**Background:**

Respiratory syncytial virus (RSV) is one of the common causes of lower respiratory infections in infants. Cell lines and animals are major models for studying RSV, but single cell type and species differences make them difficult to mimic human physiological features. Stem cell-derived organoids contain multiple cell types similar to native organs and model the physiological conditions of native organs.

**Methods:**

We used human lung organoids (HLOs) to investigate RSV infection. Immunofluorescent (IF) staining performed at 48 and 96 h post-infection to verified RSV target cells. Pro-inflammatory factor expression was assessed by RT-qPCR and ELISA. TUNEL+ apoptotic cells, Ki67+ proliferating cells, filopodia-like structures, and CDH1 (E-cadherin) expression were quantified at 96 h post-infection.

**Results:**

RSV-infected cells included epithelial cells and PDGFRβ+ cells. RSV induced pro-inflammatory factors and elicited significant changes in cell fate: increased TUNEL+ cells indicating enhanced apoptosis, and decreased Ki67+ cells reflecting impaired cell proliferation. Furthermore, morphological changes were evident in the infected HLOs. F-actin staining revealed a marked increase in filopodia-like structures, whereas CDH1 expression was significantly reduced. These observations indicate that RSV infection affects both cytoskeletal integrity and cadherin-mediated cell junctions in HLOs.

**Conclusion:**

Our findings confirm HLOs provide a valuable model for studying RSV infection *in vitro*. HLOs infected with RSV have a similar cell distribution to native lung and provide evidence of actin cytoskeleton remodeling and impaired cell adhesion.

## 1 Introduction

Respiratory syncytial virus (RSV) is a major pathogen responsible for upper and lower respiratory tract infections, contributing to increased morbidity and mortality in children (1). Severe RSV infection results in necrosis, epithelial cell shedding, and peribronchiolar inflammation leading to shortness of breath and respiratory failure (2). The prevalence of RSV infection surged in 2021 following the coronavirus disease 2019 (COVID-19) pandemic, contributing to the global burden of this disease (3).

Generally, cell lines and animals are major models to study RSV infection and injury. These models have enormously enriched our understanding of RSV infection and pathogenesis. However, traditional two-dimensional (2D) cell lines do not fully simulate the complex *in vivo* environment. Additionally, species differences prevent animal models from accurately reproducing the natural biological processes after RSV infection in humans (4). The air–liquid interface (ALI) system mimics *in vivo* airway epithelium and contains several epithelial cell types—ciliated cells, goblet cells, secretory cells, and basal cells (5)—whereas the ALI system usually lacks mesenchymal, alveolar, and endothelial cells, thereby limiting the information obtainable from RSV infection studies.

In recent years, human-induced pluripotent stem cell (hiPSC) and human embryonic stem cell (hESC)-derived human lung organoids (HLOs) have been shown to possess different cell types (6), such as ciliated cells, club cells, basal cells, and goblet cells. Although HLOs do not include the vascular system, immune cells, and nervous system, airway cell populations are intrinsic components of HLOs and functionally relevant to RSV infection. For instance, mesenchymal cells in HLOs have been shown to exhibit an altered expression of key molecules such as the TRPV1 calcium channel following RSV exposure, highlighting their role in mediating host–virus interaction (7). Owing to their physiological relevance and multicellular complexity, HLOs have already been used to study RSV infection and injury (7, 8).

Our study generated HLOs containing lung epithelial cells (ciliated cells, basal cells, club cells, goblet cells, AT1 cells, and AT2 cells), mesenchymal cells, and vascular progenitors (KDR+). Immunofluorescent staining (IF) results indicated that ciliated cells (Ace-tubulin+), alveolar type 2 (AT2) cells (Pro-SPC+), club cells (CC10+), and mesenchymal cells (PDGFRβ+) were susceptible to RSV. Quantitative real-time polymerase chain reaction (RT-qPCR) and enzyme-linked immunosorbent assay (ELISA) results showed that infected HLOs upregulated IL-8, IL-6, and IFN-β, mimicking *in vivo* innate immune response upon RSV infection. In addition, RSV infection induced apoptosis and inhibited cell proliferation. Furthermore, RSV infection promoted filopodia-like structures and reduced E-cadherin expression. Collectively, these results suggest that HLOs are a valuable model for studying RSV infection and associated epithelial injury.

## 2 Materials and methods

### 2.1 Cells and viruses

hiPSCs were purchased from ATCC (Cat#ACS-1007) and were maintained in Matrigel (BD Biosciences, Cat#354277) in mTeSR1 medium (STEMCELL Technologies, Cat#85850). The use of hiPSCs was approved by Ethics Committees of Guizhou Provincial People’s Hospital (approval number: 2023136). The RSV/A strain was isolated from nasopharyngeal secretions, and the procedures for sample collection have been described in a previous study (9). The use of RSV/A was approved by the Ethics Committees of Guizhou Provincial People’s Hospital (approval number: 202044).

### 2.2 HLO generation

HLOs were generated according to protocols described in previous studies (10, 11). Briefly, hiPSCs were treated with 100 ng/mL Activin A (R&D Systems, Cat#338-AC-050) and 2 μmol/L CHIR99021 (Tocris Bioscience, Cat#4423-10) to induce the definitive endoderm (DE) stage. The DE cells were incubated in Advanced DMEM/F12 medium (Life Technologies, Cat#12634010) with 200 ng/mL Noggin (R&D Systems, Cat#6057-NG-100), 10 μmol/L SB431542 (Tocris Bioscience, Cat#1614-10MG), 500 ng/mL FGF4 (PeproTech, Cat#100-31-250UG), 1% NEAA (Life Technologies, Cat#11140050), 1% GlutaMAX (Life Technologies, Cat#35050061), 1% B27 (Life Technologies, Cat#17504044), 1% N2 supplement (Life Technologies, Cat#A1370701), and 2 μmol/L CHIR-99021 (Tocris Bioscience, Cat#4423-10MG) to induce the anterior foregut endoderm (AFE) stage. AFE cells were embedded in 3D Matrigel (BD Biosciences, Cat#356237) with lung organoid medium containing 1% FBS, 1% NEAA, and 1% GlutaMAX to generate HLOs.

### 2.3 RSV infection

RSV/A was used in these experiments. HLOs were released from Matrigel and incubated with RSV (MOI = 0.2) for 4 h at 37°C in lung organoid medium. HLOs were washed three times to remove unbound viruses and cultured in suspension in lung organoid medium for 4 days. The supernatant was collected, and organoids were resuspended in 500 μL TRIzol reagent (Ambion, Cat#15596026). The supernatant RNA was extracted using a QIAamp Viral RNA Mini Kit (Qiagen, Cat#52904) following the manufacturer’s protocol. Work with infectious viruses was performed in BSL-2.

### 2.4 RT-qPCR

cDNA was synthesized using 1 μg total RNA with RT Master Mix for qPCR II (MCE, Cat#HY-K0511A). cDNA was diluted 1:5 in RNAse-free water and used as a template for RT-qPCR using the Talent qPCR premix following the manufacturer’s protocol (TIANGEN, Cat#FP209-02) with primers (**Table 1**). RT-qPCR was performed on a Bio-Rad CFX96 system. Genes expression was normalized to GAPDH and compared with those in hiPSCs using the 2^−ΔΔCT^ method. Viral RNA copy numbers were determined according to the standard curve method. Three or more biological replicates were performed for each assay, and the data bars represent mean ± SD.

**Table 1:** Primers used in present study.

Gene	Forward primer (5′ to 3′)	Reverse primer (5′ to 3′)
GAPDH	ACAACTTTGGTATCGTGGAAGG	GCCATCACGCCACAGTTTC
SOX2	TACAGCATGTCCTACTCGCAG	GAGGAAGAGGTAACCACAGGG
SOX17	GTGGACCGCACGGAATTTG	GGAGATTCACACCGGAGTCA
P63	CCACCTGGACGTATTCCACTG	TCGAATCAAATGACTAGGAGGGG
MUC5AC	ACCAATGCTCTGTATCCTTCCC	GTTTGGGTGGAGTAAGCCACA
NKX2.1	CTCATGTTCATGCCGCTC	GACACCATGAGGAACAGCG
SFTPC	AGCAAAGAGGTCCTGATGGA	CGATAAGAAGGCGTTTCAGG
HOPX	GCCTTTCCGAGGAGGAGAC	TCTGTGACGGATCTGCACTC
PDGFRβ	AGCACCTTCGTTCTGACCTG	TATTCTCCCGTGTCTAGCCCA
SCGB1A1	TTCAGCGTGTCATCGAAACCC	ACAGTGAGCTTTGGGCTATTTTT
RSV N	CACAGAAGATGCAAATCATAAATTCA	GTATCTTTATGGTGTCTTCTCTTCCTAACC
IL-8	ACTGAGAGTGATTGAGAGTGGAC	AACCCTCTGCACCCAGTTTTC
IL-6	ACTCACCTCTTCAGAACGAATTG	CCATCTTTGGAAGGTTCAGGTTG
IL-1β	AGCTACGAATCTCCGACCAC	CGTTATCCCATGTGTCGAAGAA
TLR3	TCTGGAAACACGCAAACCCT	GGGATCTCGTCAAAGCCGTT

### 2.5 IF staining

Samples were treated with 4% paraformaldehyde overnight at 4 °C. The organoids were rinsed three times with PBST (PBS (Solarbio, Cat#P1010) containing 0.1% Tween 20 (Solarbio, Cat#IT9010)). The samples were embedded in 3D Matrigel, covered with O.C.T. compound cryostat embedding medium (SciGen, 4583), and frozen at −80 °C. These samples were washed with PBST three times and subsequently permeabilized with 0.4% Triton X-100 (Solarbio, Cat#T8200). After 40 min at room temperature (RT), the samples were washed with PBST and blocked with 5% BSA at RT for 2 h. The samples were then incubated with primary antibodies overnight at 4 °C. Subsequently, they were washed and stained with PBST and incubated with secondary antibodies at RT for 1 h. Nuclei were counterstained with Hoechst (Invitrogen, Cat#H3570) for 3 min and then washed twice with PBST. VECTASHIELD antifade mounting medium was added (Vector Laboratories, Cat#H-1000-10) before covering with glass microscope slides. Imaging was performed on a Carl ZEISS LSM 980 system and analyzed with ZEN 2 software. Antibodies used in the present study are listed in **Table 2**.

**Table 2:** Antibodies used in present study.

Antibodies	Host	Dilution	Manufacture	Catalog number
NKX2.1	Rabbit	1:200	Abcam	Ab76013
NKX2.1	Rabbit	1:200	Cell Signaling Technology	12373S
P63	Rabbit	1:200	Abcam	Ab12462
MUC5AC	Rabbit	1:200	Abcam	EPR16904
CC10	Rabbit	1:200	Invitrogen	MA5-34625
Ace-tubulin	Mouse	1:600	Sigma	T7451
PDPN	Hamster	1:200	Invitrogen	MA5-16113
Pro-SPC	Rabbit	1:200	Abcam	Ab211326
PDGFRβ	Rabbit	1:200	HUABIO	ET1605-20
KDR	Rabbit	1:200	Cell Signaling Technology	2479S
Ki67	Rabbit	1:200	Abcam	Ab16667
Actin-Tracker Red-555		1:200	Beyotime	C2203S
CDH1	Rabbit	1:200	Abcam	Ab40772
RSV Polyclonal Antibody, FITC (2F7)	Mouse	1:200	Santa Cruz Biotechnology	sc-101362
RSV Polyclonal Antibody, FITC	Goat	1:200	Invitrogen	PA1-73017
Donkey anti-rabbit (Alexa 555)	Donkey	1:500	Invitrogen	Ab32794
Donkey anti-mouse (Alexa 488)	Donkey	1:800	Invitrogen	Ab32766
Donkey anti-mouse (Alexa 555)	Donkey	1:500	Invitrogen	A32773
Donkey anti-hamster (Alexa 568)	Goat	1:500	Invitrogen	A21112

### 2.6 TUNEL staining

A one-step TUNEL *in situ* apoptosis kit (Elabscience, Cat#E-CK-A320) was performed according to the manufacturer’s protocol. The samples were equilibrated to RT, immersed in 4% Triton X-100, and incubated at RT for 40 min. The samples were washed with PBS twice for 5 min (per wash). 100 μL of 1× Proteinase K working solution was added to each sample, incubated at 37 °C for 20 min, and washed with PBS three times for 5 min (per wash). 100 μL TdT Equilibration Buffer was added to each sample and incubated at 37 °C for 30 min. After this, 50 μL Labeling Working Solution was added to each sample and incubated in the dark (using a humidified light-excluding chamber) at 37 °C for 1 h. The samples were washed with PBS three times for 5 min (per wash) prior to adding diluted Hoechst solution and incubated at RT for 4 min in a light-tight box. The samples were washed with PBS four times for 5 min (per wash) before adding an antifade mounting medium to seal the slides. Imaging was performed on a Carl ZEISS LSM 980 system.

### 2.7 ELISA

Cytokine detection was performed using ELISA kits (Elabscience, E-EL-H6156, E-EL-H6008, and E-EL-H0085) following the manufacturer’s protocol. 100 μL per well of the prediluted standard, blank, and sample was plated before sealing. The plates were incubated for 90 min at 37 °C before the solution was decanted from each well and replaced with 100 μL Biotinylated Detection Ab working solution. The plates were incubated for 1 h at 37 °C, and the solution from each well was removed, replaced with 350 μL wash buffer, and dried. 100 μL of HRP conjugate working solution was added to each well and incubated for 30 min at 37 °C. Wells were decanted, washed, and dried before adding 90 μL Substrate Reagent to each well. After a 20-min incubation at 37 °C (protected from light), 50 μL of Stop Solution was added to each well and read using a 37°C preheated Biotek Synergy H1 system to determine the optical density (OD value) at 450 nm and analyzed with ELISACalc.

### 2.8 Statistical analysis

GraphPad Prism 9.5 was used to analyze the experimental data. Normality and homogeneity of variance were tested before statistical analysis. Independent-samples t-test was used for comparison between two groups, whereas one-way ANOVA with Tukey’s *post-hoc* test was applied for comparisons among three or more groups. Mean ± SD noted, *, *P* < 0.05, **, *P* < 0.01, ***, *P* < 0.001, and ****, *P* < 0.0001.

## 3 Results

### 3.1 Generation of HLOs from hiPSCs

**Figure 1:**
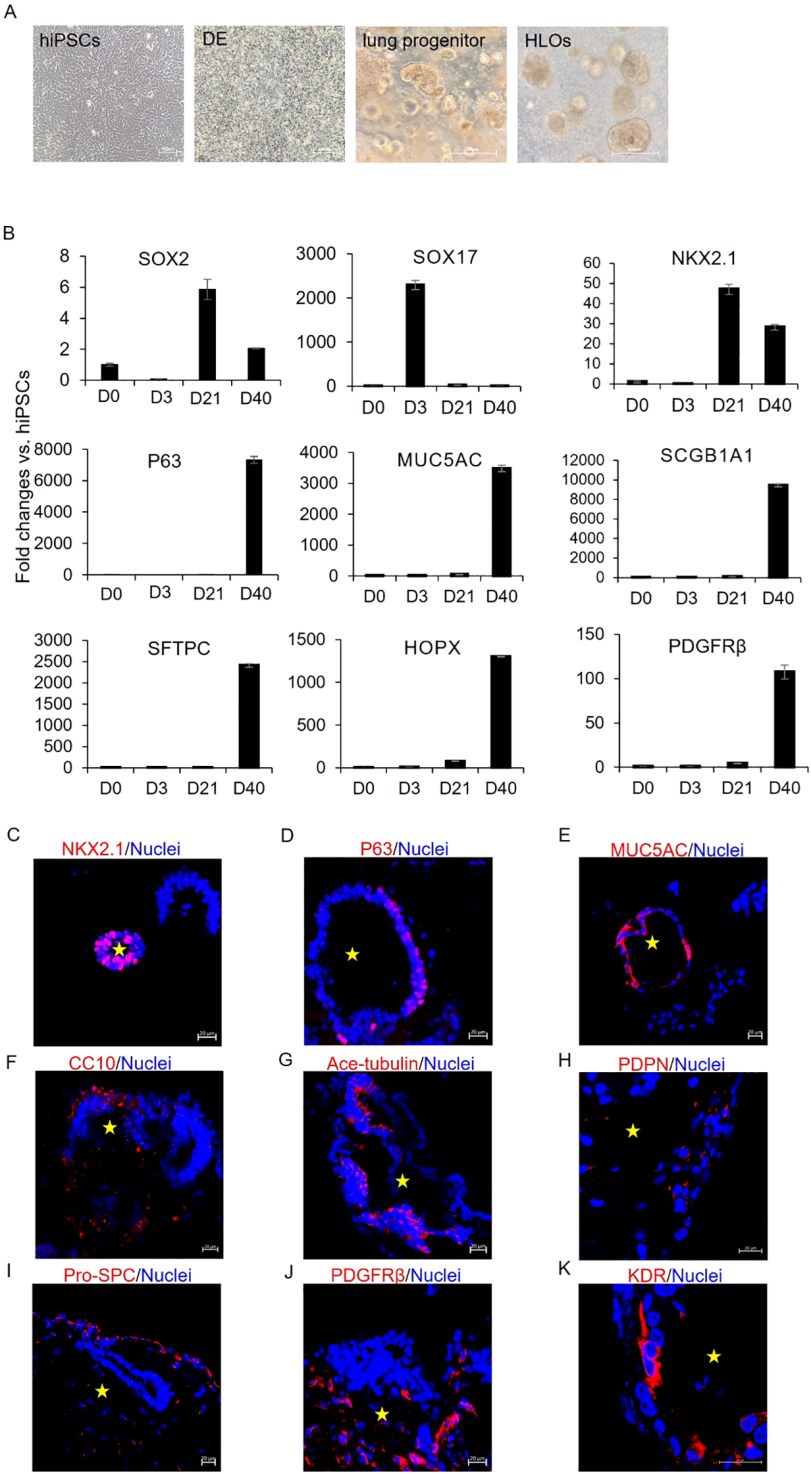
Generation of HLOs derived from hiPSCs. **(A)** Brightfield field for hiPSCs (day 0), DE (day 3), lung progenitor organoids (day 21), and mature stage HLOs (day 40), scale bar: 100 μm. **(B)** Fold change of lineage marker genes from day 0 to day 40 over undifferentiated hiPSCs by quantitative RT-qPCR (2−ΔΔCt). hiPSCs and proximal tracheal progenitor cell (*SOX2*), definitive endoderm marker (*SOX17*), lung progenitor cells (*NKX2.1*), basal cell (*P63*), goblet cell (*MUC5AC*), club cell (*SCGB1A1*), AT2 cells (*SFTPC*), AT1 cells (*HOPX*), and mesenchymal cell (*PDGFRβ*). Normalized to GAPDH (N = 3). Data are presented as mean ± SD, and statistical analysis was assessed using one-way ANOVA. **(C–K)** IF staining of lung epithelial cells: lung progenitor cells (NKX2.1), basal cells (P63), goblet cells (MUC5AC), club cells (CC10), ciliated cells (Ace-tubulin), AT1 cells (PDPN), AT2 cells (Pro-SPC), mesenchymal cells (PDGFRβ), and vascular progenitor (KDR) at day 40. Nuclei staining with Hoechst. The asterisk indicates the lumen of organoids. Scale bar: 20 μm.

Lung development progresses through a series of stages: from the definitive endoderm (DE), anterior foregut endoderm (AFE), and lung progenitor to the mature lung. Brightfield microscopy revealed morphological changes in cells during stepwise HLO induction (**Figure 1A**). By day 40, epithelial cells had existed into well-formed 3D spherical HLOs with distinct, stable morphology. RT-qPCR analysis revealed the temporal expression dynamics of differentiation marker genes. *SOX17* was upregulated during the DE stage, whereas *SOX2* and *NKX2.1* were upregulated in the lung progenitor stage. As HLO differentiation progressed, markers of epithelial cell types, including *P63*, *SCGB1A1*, *MUC5AC*, *SFTPC*, *HOPX*, and *PDGFRβ* showed gradual increases in expression in mature stage HLOs (**Figure 1B**).

In the early stage of differentiation, lung progenitor cell marker NKX2.1 was expressed (**Figure 1C**). Additionally, IF staining revealed the presence of epithelial cell markers in day 40 HLOs, including ciliated cells (Ace-tubulin+) with cilia existing in the lumen and outer layer of HLOs, basal cells (P63+), club cells (CC10+), goblet cells (MUC5AC+), alveolar type 2 cells (AT2) (Pro-SPC+), and alveolar type 1 cells (AT1) (PDPN+) (**Figures 1D–I**). Beyond lung epithelium, PDGFRβ was detected in HLOs, confirming the presence of PDGFRβ+ mesenchymal cells (**Figure 1J**). Furthermore, a small population of differentiating cells expressed KDR (**Figure 1K**), a marker of vascular progenitors. However, these KDR+ cells failed to form vessel-like structures.

### 3.2 RSV infects lung epithelial cells and mesenchymal cells in HLOs

**Figure 2:**
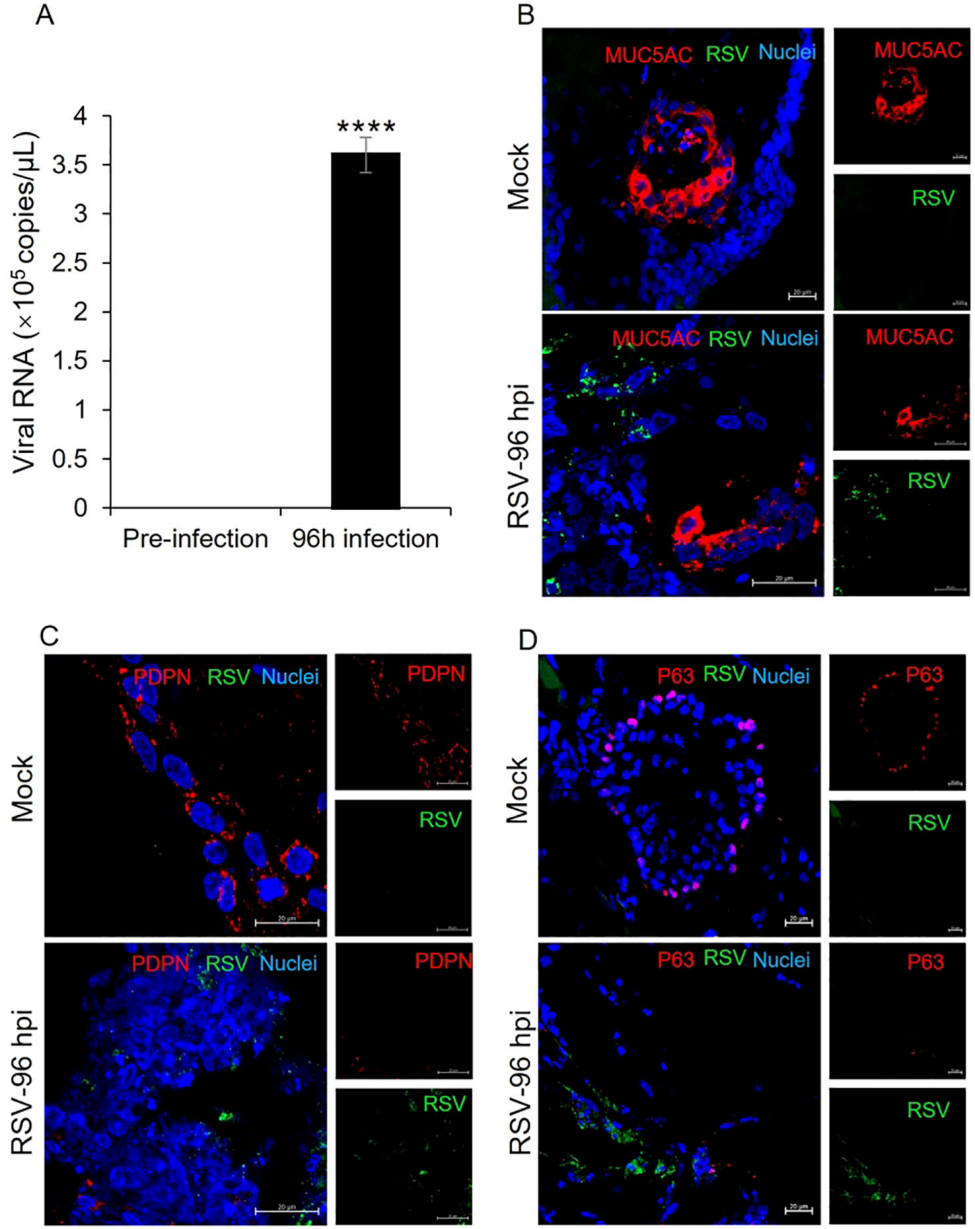
Infection of HLOs with RSV. **(A)** Comparison of RSV N mRNA expression in HLOs pre- and 96 h post-RSV infection was determined by RT-qPCR assays (N = 3). Data are presented as mean ± SD. and statistical significance was assessed using a two-tailed t-test. ****, P < 0.0001. **(B–D)** Confocal immunofluorescence microscopy of cells within RSV-infected HLOs (MOI = 0.2) post-96 h infection: RSV F and epithelial cell lineage marker expression. Goblet cells (MUC5AC), AT1 cells (PDPN), and basal cells (P63). Scale bars: 20 μm.

**Figure 3:**
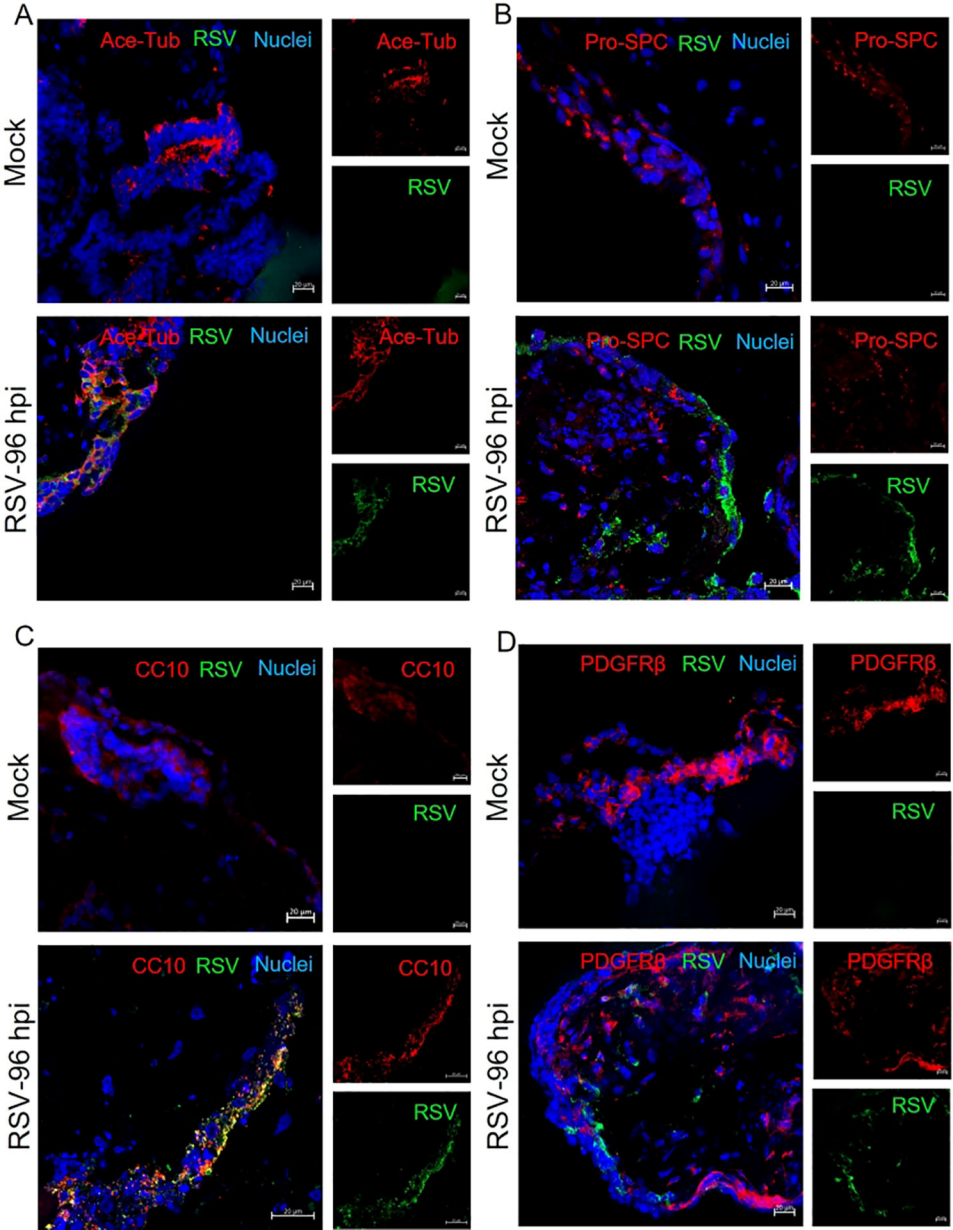
Infection of HLOs with RSV for 96 h. **(A–D)** Confocal immunofluorescence microscopy of cells within RSV-infected HLOs (MOI = 0.2) post-96 h infection: RSV F and epithelial cell lineage marker expression. Ciliated cells (Ace-tubulin), AT2 cells (Pro-SPC), club cells (CC10), and mesenchymal cells (PDGFRβ). Scale bars: 20 μm.

**Figure 4:**
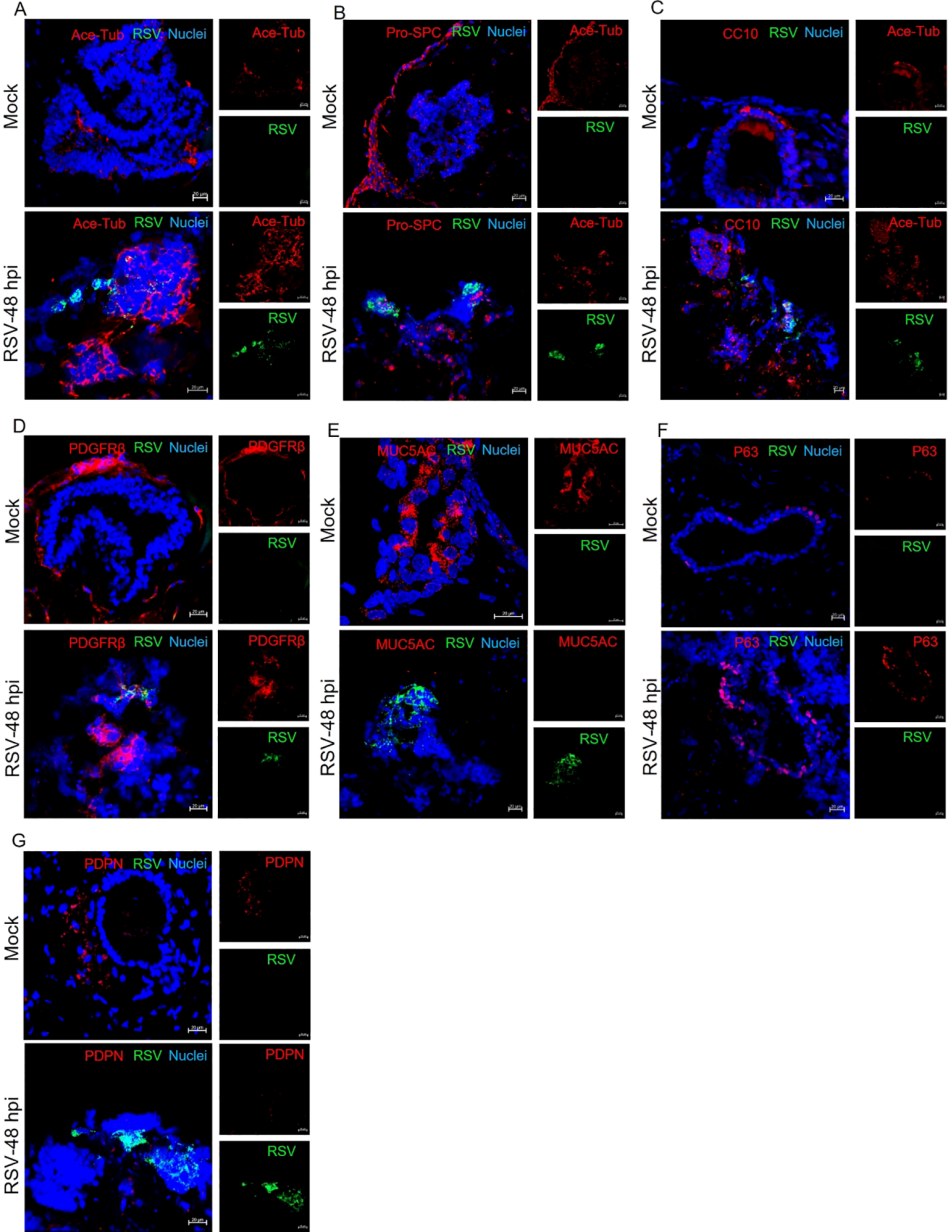
Infection of HLOs with RSV for 48 h. **(A–G)** Confocal immunofluorescence microscopy of cells within RSV-infected HLOs (MOI = 0.2) post-48 h infection: RSV F and epithelial cell lineage marker expression. Ciliated cells (Ace-tubulin), AT2 cells (Pro-SPC), club cells (CC10), and mesenchymal cells (PDGFRβ) were susceptible to RSV, goblet cells (MUC5AC), basal cells (P63) and AT1 cells (PDPN) were not susceptible to RSV. Scale bars: 20 μm.

Next, day 40 HLOs were exposed to RSV (MOI = 0.2) to identify the cellular distribution of RSV infection. RT-qPCR results showed the transcriptional level of RSV mRNA was significantly elevated, indicating efficient viral replication in the infected HLOs during the RSV culture period (**Figure 2A**). Then, we performed co-staining for lung epithelial markers and RSV F protein to determine which cell types were infected. IF staining results showed that no RSV+ cells were observed in goblet cells (MUC5AC+), basal (P63+), and AT1 cells (PDPN+) (**Figures 2B–D**). Further analysis revealed that RSV-infected ciliated cells (Ace-tubulin+) and AT2 cells (Pro-SPC+) (**Figures 3A, B**). Among RSV+ cells, ciliated cells accounted for the highest proportion (**Table 3**). Additionally, club cells (CC10+) and mesenchymal cells (PDGFRβ+) were also susceptible to RSV (**Figures 3C, D**). RSV F protein was detected in epithelial cells (Ace-tubulin+, CC10+, Pro-SPC+), and mesenchymal cells (PDGFRβ+) distributed across different regions of HLOs at both 48 hpi (**Figure 4**) and 96 hpi (**Figure 3**). Infected epithelial cells were observed in luminal-facing structures and more peripheral regions, whereas infected mesenchymal cells were located in deeper, sub-epithelial areas. Since only a small population of KDR+ vascular progenitors was observed in HLOs, whether RSV could infect these cells remains unknown.

**Table 3:** Cell composition of RSV-positive cells.

Cell type	Marker/RSV
AT1 (PDPN)	0/416 (0%)
AT2 (Pro-SPC)	89/416 (21.4%)
Basal cell (p63)	0/416 (0%)
Club cell (CC10)	35/416 (8.4%)
Goblet cell (MUC5AC)	0/416 (0%)
Ciliated cell (Ace-tubulin)	187/416 (45%)
Mesenchymal cell (PDGFRβ)	105/416 (25.2%)

Percentages indicate the proportion of each marker-positive cell type among all RSV F-positive cells within HLOs. Marker and Hoechst-positive cells were counted in randomly selected view fields within a section.

### 3.3 RSV-infected HLOs mimic several aspects of lung injury

**Figure 5:**
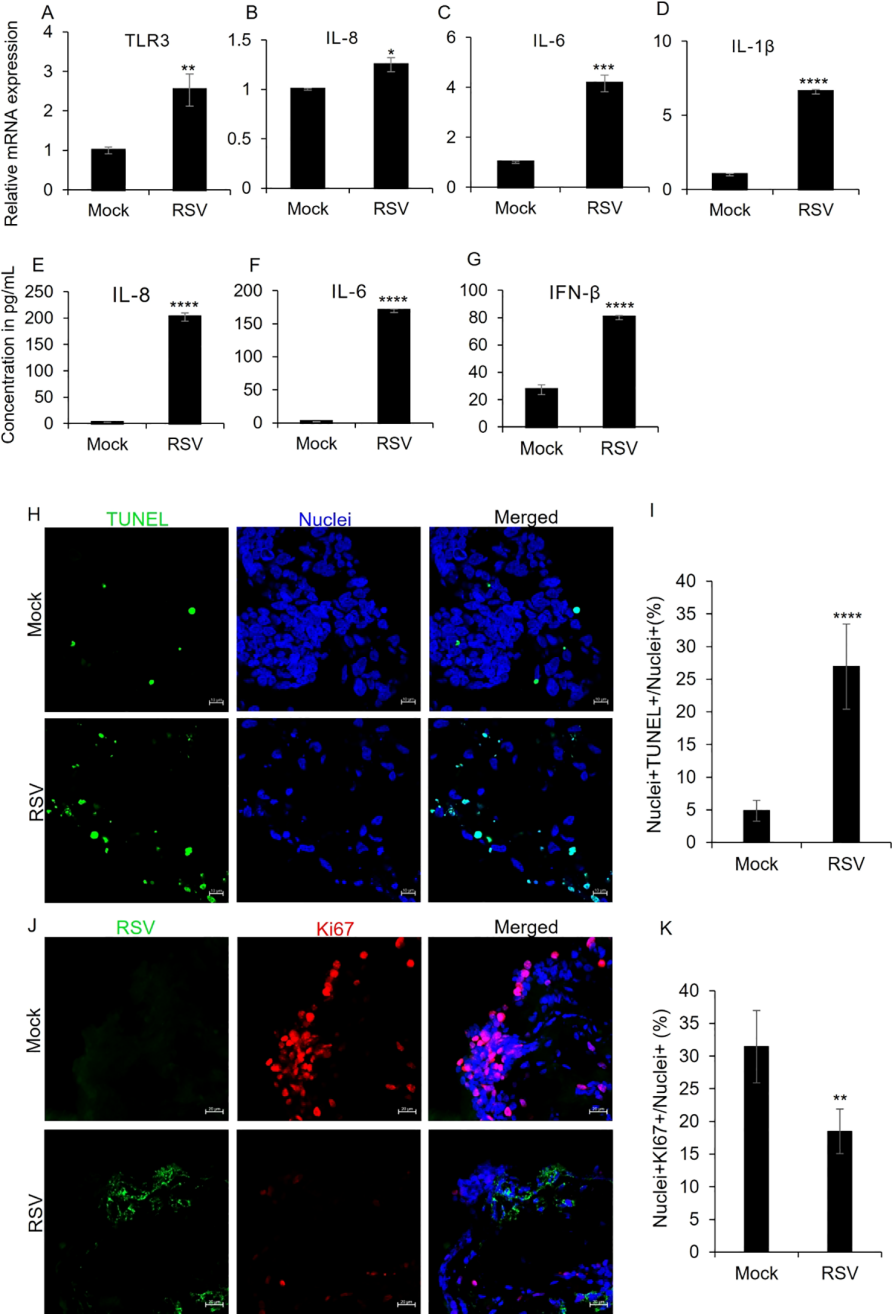
Immune cytokine/chemokine profile and apoptosis for HLOs after RSV infection. **(A–D)** Quantifying TLR3, IL-8, IL-6, and IL-1β for RSV-induced HLOs using RT-qPCR (N = 3). **(E–G)** IL-8, IL-6, and IFN-β ELISA profile for RSV-infected HLOs (N = 4). **(H)** TUNEL staining for RSV-infected HLOs and the mock group. **(I)** A statistical analysis for the percentage of apoptotic cells in RSV-infected HLOs and the mock group (N = 6). **(J)** IF staining for Ki67 in RSV-infected HLOs and the mock group. **(K)** Statistical analysis for the percentage of proliferative cells in RSV-infected HLOs and the mock group (N = 5). Scale bars: 10 μm. Data are presented as mean ± SD, and statistical significance was assessed using a two-tailed t-test. *, *P* < 0.05, **, *P* < 0.01, ***, *P* < 0.001, and ****, *P* < 0.0001.

Toll-like receptors (TLRs) sense viral particles and virus-derived nucleic acids during RSV infection (12). RT-qPCR confirmed the upregulation of TLR3 expression in RSV-infected HLOs (**Figure 5A**). Next, we confirmed that cytokine expression was elevated in RSV-infected HLOs compared with uninfected controls (**Figures 5B, C**). Additionally, our data showed that IL-1β mRNA expression was upregulated after RSV infection (**Figure 5D**). Furthermore, ELISA assays revealed that IL-8 increased 82-fold, IL-6 increased 26-fold, and interferon-β (IFN-β) increased 3-fold 96 h post-infection (**Figures 5E–G**).

**Figure 6:**
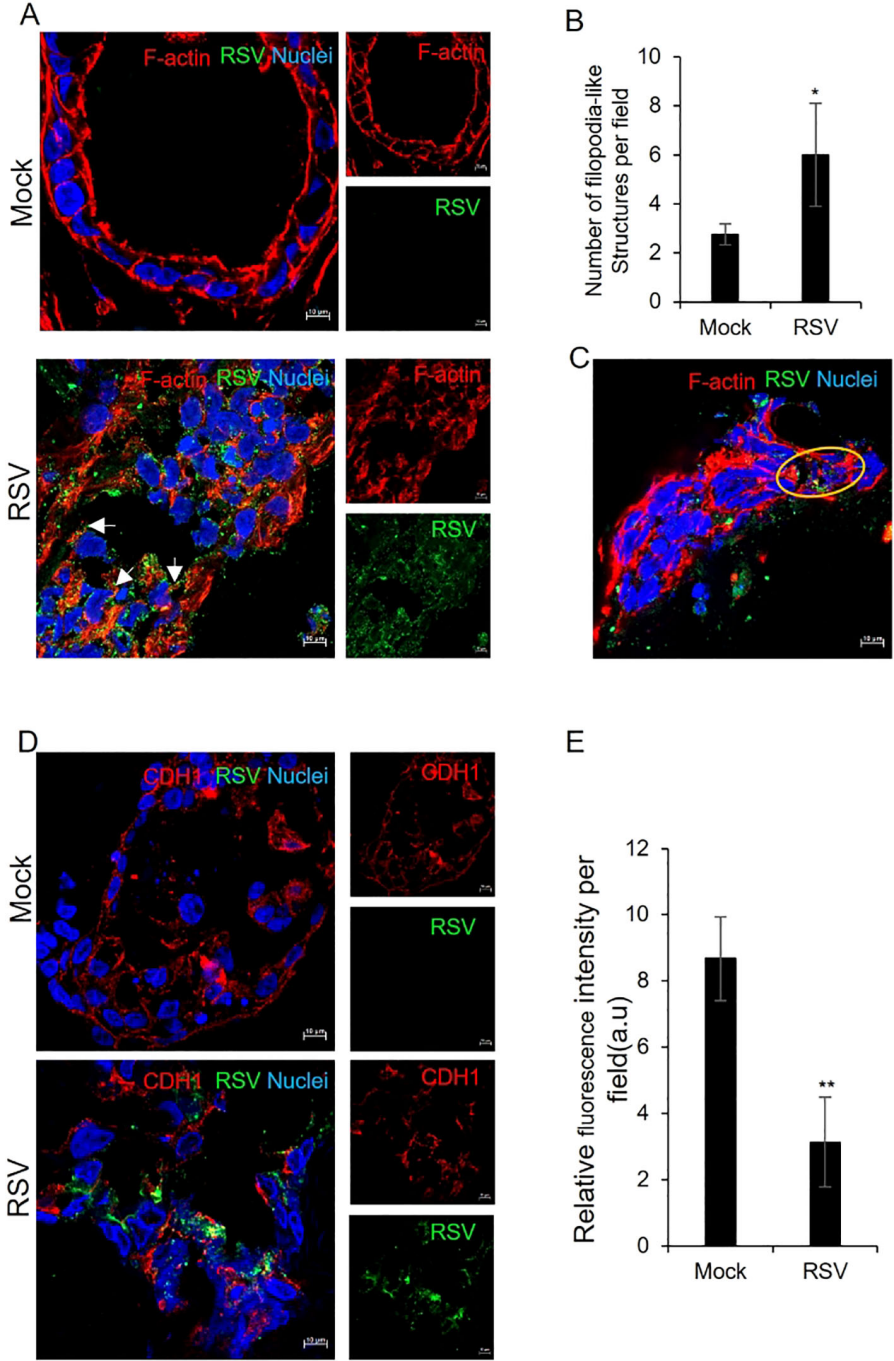
RSV infection affects F-actin structure and E-cadherin (CDH1) expression. **(A)** IF for F-actin in RSV-infected HLOs and the mock group 96 h post-infection. White arrowheads pointing to filopodia-like structures. **(B)** Statistical analysis for the number of filopodia-like structures in HLOs (N = 4). **(C)** IF of RSV-infected HLOs showing syncytia formation. Scale bars: 10 μm. **(D)** IF for CDH1 in RSV-infected HLOs and mock group. **(E)** Statistical analysis for the relative fluorescence intensity of CDH1 (N = 3). Scale bars: 10 μm. Data are presented as mean ± SD, and statistical significance was assessed using a two-tailed t-test. *, P < 0.05, **, P < 0.01.

Apart from immune responses, programmed cell death is another way host cells protect themselves (13). We verified apoptosis in HLOs upon RSV infection. TUNEL staining of RSV-infected HLOs showed that the structure of RSV-infected HLOs was affected compared with the mock group, and apoptosis increased approximately 5-fold (**Figures 5H, I**). Meanwhile, IF staining showed that the Ki67 proliferation marker was reduced in RSV-infected HLOs (**Figures 5J, K**). The actin cytoskeleton and adherens junctions are essential for maintaining epithelial barrier function and epithelial homeostasis. RSV infection increased the number of filopodia-like structures (**Figures 6A, B**). Moreover, syncytia were also observed (**Figure 6C**). E-cadherin (CDH1), an important protein for maintaining lung epithelial integrity, was downregulated in RSV-infected HLOs (**Figures 6D, E**). These results indicate that RSV infection can induce an innate immune response and apoptosis, inhibit proliferation, promote filopodia-like structures, and downregulate E-cadherin in HLOs.

## 4 Discussion

This study generated HLOs to investigate RSV infection patterns and epithelial injury. The native lung contains multiple types of epithelial, mesenchymal, immune, and vascular cells (14). In the present study, HLOs contained epithelial cells, mesenchymal cells, and a small population of KDR+ vascular progenitors. Previous studies have successfully established vascularized human lung progenitor organoids and adopted vascular induction cocktails to construct spatially organized and branched vascularized cardiac organoids (15, 16). Nevertheless, no study has yet generated HLOs containing vascular endothelial cells via the differentiation of hiPSCs. Therefore, we hypothesize that co-differentiating hiPSCs can be applied to generate HLOs with vascular endothelial cells, providing a more physiologically relevant model for investigating RSV infection *in vitro*.

In human lung, RSV is frequently detected in ciliated cells (17), and infected ciliated cells lacked cilia similar to those found in murine RSV-infection models with human lung implants (18). Consistent with previous studies (19–23), our results showed that ciliated cells (Ace-tubulin+), club cells (CC10+), and AT2 (Pro-SPC+) cells are susceptible to RSV, whereas basal (P63+) and goblet cells (MUC5AC+) are not observed. Mesenchymal cells are crucial in lung development and maintain physiological homeostasis. Harford et al. demonstrated that RSV can infect α-SMA+ and vimentin+ cells in HLOs (7). We further observed that PDGFRβ+ mesenchymal cells display susceptibility to RSV infection. However, as we did not perform co-staining with other mesenchymal lineage markers on PDGFRβ+ cells, the subsets of PDGFRβ+ cells susceptible to RSV remain to be defined. Further studies are therefore required to identify the exact mesenchymal cell subtypes vulnerable to RSV.

Innate immunity is the line of defense against viral infections. We observed an increased expression of TLR3 in RSV-infected HLOs. TLRs bind pathogen-related molecular patterns and activate transcription factors to upregulate antiviral and pro-inflammatory cytokines (24), such as IL-8 and IL-6 which were selected as well-established biomarkers of RSV-induced innate immunity (25). We demonstrated that RSV-infected HLOs exhibited increased secretion of IL-8 and IL-6, consistent with previous studies (19). The elevated production of these inflammatory cytokines reflects a typical host inflammatory response triggered by RSV infection but does not fully recapitulate complex immune cell recruitment seen *in vivo*.

TUNEL staining verified that apoptosis in RSV-infected HLOs increased. Welliver et al. (26) demonstrated that RSV increases bronchiolar epithelial cell death in fetal lung tissue. Apoptosis, which has been associated with less severe RSV infection in children, is thought to be related to better control of viral replication (27). In infant-derived nasal organoids, the level of cell death quantified by Caspase 3/7 activity was also increased upon RSV infection (28). Meanwhile, human bronchial epithelial cells are arrested in the G1 and G2/M phases of the cell cycle after RSV infection (29), suggesting that RSV may affect cell proliferation. Our results demonstrate that the number of Ki67+ proliferative cells decreased in RSV-infected HLOs. However, there were no statistically significant changes in the amount of cell proliferation in RSV/A or RSV/B-infected HNO-ALI (28). Whether such proliferative differences result from variations in viral titers or distinct experimental models requires further study. Collectively, these results indicate that HLOs can model the process of RSV infection, including the innate immune response, cell apoptosis, and cell proliferation.

Filopodia and syncytia are the core mechanisms of RSV cell-to-cell transmission (30). Syncytia are multinucleated structures induced by RSV F protein (31), allowing cytoplasmic exchange and rapid viral spread. This cell–cell fusion process is partially shielded from extracellular neutralizing antibodies (32). Concurrently, RSV induces host cells to form filopodia, creating direct channels for virus particle transport (33). F-actin composed filopodia enhance the uptake of virus by airway epithelial cells, and promote RSV invasion and intercellular transmission (34). In the present study, our IF staining results clearly captured these two mechanisms (**Figures 6A, C**), confirming active viral protein expression during membrane fusion. These mechanisms act synergistically: Filopodia mediate the delivery of viral particles to neighboring cells, whereas syncytia enable dissemination within the epithelial layer through direct cell-cell fusion.

The formation and function of filopodia depend on the actin skeleton and apical junction complexes (AJCs) of host cells, which are key to maintaining epithelial homeostasis and barrier function (35). E-cadherin mediates several physiological processes, including cell–cell adhesion, proliferation, and differentiation (36). There have been conflicting reports on the expression of E-cadherin after RSV infection. For example, OVA-RSV-challenged mice exhibited E-cadherin loss, and E-cadherin expression decreased in RSV-infected NCI-H292 cells (37, 38). While RSV infection did not change E-cadherin expression in A549 and normal human bronchial epithelial cells (NHBECs), it was shown to be increased in NHBECs cultured in a differentiating ALI system (39). Notably, RSV infection decreased E-cadherin expression in the present study. A key difference between the A549, NHBEC, and ALI systems and the HLOs in our study is that HLOs incorporate not only lung epithelial cells but also mesenchymal cells and a complex microenvironment. However, whether this complex structure and physiological environment promote the degradation of E-cadherin after RSV infection remains to be further studied. By utilizing cell structures closely related to epithelial barrier function, RSV can efficiently achieve cell-to-cell transmission, which not only avoids extracellular immune surveillance but also relies on the host’s own molecular mechanisms to enhance the spread of infection, providing support for understanding how RSV controls host cellular components to mediate pathogenesis. In this regard, our HLOs may provide valuable biological insights into RSV infection.

However, this study also has several limitations. Cilia were observed on both luminal and basal-facing surfaces. It is difficult to distinguish whether RSV entry cells through apical or basolateral side. Although HLOs better simulate physiological characteristics than traditional monolayer cell models, and our HLO model contains only a small population of KDR+ vascular progenitors, their microenvironment is far less complex than that of natural human lung tissue. Moreover, the model lacks several critical components of native lung tissue, including immune cells like macrophages, dendritic cells, and lymphocytes, as well neural cells. This restricts the study of immune-mediated pathogenesis and neuro-epithelial interactions during RSV infection. In addition, IF analysis using frozen sections can only reflect the status of specific cell populations at a single time point, lacking dynamic observation.

In the future, we will optimize culture conditions to establish stable apical–basal polarity and explore the approach of co-differentiation of HLOs and vascular structures. This will help to evaluate the sensitivity of endothelial cells to RSV. In addition, single-cell multi-omics and immune-biomarker frameworks that have been used in inflammatory diseases may help comprehensively characterize cell heterogeneity and determine RSV-targeted cell types.

## Data Availability

The raw data supporting the conclusions of this article will be made available by the authors, without undue reservation.
